# Increased neural noise and impaired brain synchronization in fibromyalgia patients during cognitive interference

**DOI:** 10.1038/s41598-017-06103-4

**Published:** 2017-07-19

**Authors:** A. J. González-Villar, N. Samartin-Veiga, M. Arias, M. T. Carrillo-de-la-Peña

**Affiliations:** 1 Departamento de Psicoloxía Clínica e Psicobioloxía, Facultade de Psicoloxía, Universidade de Santiago de Compostela, Santiago de Compostela, Spain; 2 Departamento de Neurología, Complexo Hospitalario Universitario de Santiago, Santiago de Compostela, Spain

## Abstract

Fibromyalgia (FM) and other chronic pain syndromes are associated with cognitive dysfunction and attentional deficits, but the neural basis of such alterations is poorly understood. Dyscognition may be related to high levels of neural noise, understood as increased random electrical fluctuations that impair neural communication; however, this hypothesis has not yet been tested in any chronic pain condition. Here we compared electroencephalographic activity (EEG) in 18 FM patients -with high self-reported levels of cognitive dysfunction- and 22 controls during a cognitive control task. We considered the slope of the Power Spectrum Density (PSD) as an indicator of neural noise. As the PSD slope is flatter in noisier systems, we expected to see shallower slopes in the EEG of FM patients. Higher levels of neural noise should be accompanied by reduced power modulation and reduced synchronization between distant brain locations after stimulus presentation. As expected, FM patients showed flatter PSD slopes. After applying a Laplacian spatial filter, we found reduced theta and alpha power modulation and reduced midfrontal-posterior theta phase synchronization. Results suggest higher neural noise and impaired local and distant neural coordination in the patients and support the neural noise hypothesis to explain dyscognition in FM.

## Introduction

Many chronic pain patients present impaired cognitive functioning^1, 2^, as indicated by poorer performance and slower reaction times in neuropsychological tasks^3–5^. These alterations occur more frequently in patients with generalized pain^6^ such as fibromyalgia (FM), in which cognitive dysfunction has recently been recognized as one of its core symptoms^7^. Although impaired cognition may have a strong impact on quality of life, the causes of dyscognition in FM and other chronic pain pathologies are poorly understood^8^.

Cognition depends on dynamic interactions between local and large scale brain networks^9^. Disruption of these interactions by noisy and unreliable information transfer can thus cause a decline in cognitive performance^10^. The slope of the power spectral density (PSD) plot has been proposed as a useful indicator of the level of neural noise^10, 11^. In the human electroencephalogram (EEG), a reduction in power is observed as frequency increases, yielding a negative slope in the PSD plot. Synchronized neural activity is related to steeper PSD slopes, while desynchronized activity yields flatter slopes^12^ (as an extreme, a random signal such as white noise gives a flat line). This relationship between PSD slope and correlated/decorrelated neural activity has also been observed using computer simulations^13, 14^. Therefore, the slope of the linear fit of the PSD can be used to estimate the degree of noise in a system, being flatter when there is more uncoordinated activity in large neural populations^15^.

Stimulus processing in less coordinated and noisier environments may be blurred. Therefore an increase in neural noise should theoretically be accompanied by degraded time-frequency power modulation after stimulus presentation. Cognitive control and visual attention requirements are related to power modulations in midfrontal theta and posterior alpha^16, 17^. In this sense, higher noise should impair synchronization of local neural populations, reflected by reduced theta and alpha power modulation after stimulus presentation.

Cognitive dysfunction may also be related to impaired interactions between distant brain locations^18^. Thus, the predicted increase in neural noise should also be accompanied by degraded neural communication in distant-range networks. Such distant brain areas (e.g. fronto-posterior regions) are believed to be synchronized by theta or other low frequency oscillations^17, 19^.

Although the presence of heightened neural noise may be a plausible explanation for the cognitive dysfunction observed in chronic pain patients, as far as we are aware this possibility has not yet been investigated. In the present study, we analysed the slope of the PSD plot, posterior alpha and midfrontal theta power, and fronto-posterior functional connectivity (theta phase synchronization) derived from EEGs recorded while study participants performed the Multi-Source Interference Task (MSIT). The MSIT is designed to assess the integrity of cognitive/attentional networks^20^. We hypothesize that PSD slopes would be flatter for patients than for healthy controls. As convergent evidence, we also expected to find reduced modulation of midfrontal theta and posterior alpha power, along with a lower level of fronto-posterior theta phase synchronization in patients.

## Method

### Participants

In this study, we compared 19 fibromyalgia patients (FM) and 22 healthy controls (HC). The groups were matched by sex (all women), age and years of education (Table 1). The patients were initially contacted by their physicians or through patients associations, and the controls were recruited in community centres. The inclusion criteria were diagnosis of FM (usually made by a general practitioner and confirmed by a rheumatologist) and subjective complaints of cognitive dysfunction. All patients included in the study reported severe, pervasive, continuous and life-disturbing cognitive problems in the Symptom Severity Score of the Fibromyalgia Survey Questionnaire^21^. Exclusion criteria included the presence of other disease that could explain pain and fatigue, severe depression, generalized anxiety disorder or other mental disorders (but not moderate levels of depression or anxiety). The same exclusion criteria were applied to control participants, who in addition were required to not have any history of chronic pain.

**Table 1:** Mean scores and statistical comparison of the psychosocial variables measured.

	FM (n = 18)	HC (n = 22)	Difference
	mean (SD)	mean (SD)	t_38_ (p-value)
Age	43.9 (7.6)	45.1 (7.2)	0.5 (0.62)
Years of education	10.4 (3.5)	11.3 (3.3)	−0.82 (0.42)
WPI	14 (4.8)	1.7 (1.8)	10.2 (<0.001)
MFE	73.2 (22.5)	20.6 (14)	8.9 (<0.001)
BDI	25.7 (11.8)	7.13 (5.9)	6.1 (<0.001)
VAS attention	6.9 (1.7)	2.2 (2.0)	7.9 (<0.001)
VAS memory	8.0 (1.8)	2.6 (2.1)	8.5 (<0.001)
VAS concentration	7.6 (1.9)	2.5 (2.4)	7.3 (<0.001)
VAS pain	7.7 (2)	1.4 (1.6)	11 (<0.001)
VAS health	7.6 (2.3)	2.3 (2.7)	6.7 (<0.001)
VAS sleep	8.6 (2.1)	2.1 (2.4)	8.4 (<0.001)

*Abbreviations:* BDI - Beck Depression Inventory; WPI - Widespread Pain Index; MFE - Memory Failures of Everyday; VAS - Visual Analogue Scales measuring attentional, memory and concentration complaints, pain intensity, health status and quality of sleep in the last month (between 0 and 10, where 10 indicates the worst condition).

All participants were advised not to smoke or to consume coffee, alcohol or other non-medically prescribed drugs in the 2 hours prior to the evaluation. They were not asked to alter consumption of medically prescribed treatments. All subjects were paid 25 euros after the experimental session. One FM participant was excluded from the study due to inadequate performance of the task.

The experimental procedures were approved by the University of Santiago ethics committee in accordance with the Declaration of Helsinki. All the participants were informed about the experimental procedures and gave written informed consent before participation.

### Procedure and task

Participants were informed about the study procedures and provided their written consent prior to the recording session. In the clinical session, participants were interviewed about their health status and evaluated by neuropsychological tests. They also completed Visual-Analogue Scales (VAS), the Widespread Pain Index (WPI) of the Spanish version of the Fibromyalgia Survey Questionnaire (FSQ)^21^, the Beck Depression Inventory (BDI)^22^ and the Memory Failures of Everyday Questionnaire (MFE)^23^. The participants were then fitted with an EEG electrode cap and underwent the recording session in a dimly lit room.

Participants sat on a comfortable armchair in front of a 17-inch computer screen, placed at a distance of 80 cm from their gaze and then performed the Multi-Source Interference Task^20^. They were asked to respond with the right hand to the identity of the different number in a set of three numbers, while ignoring its position. The response box had 3 buttons labelled with the numbers ‘1’, ‘2’ and ‘3’ (from left to right), which participants had to press with the index, middle or ring finger. They were instructed to respond as quickly as possible without sacrificing accuracy. The instructions were clearly explained and the participants performed 10 practice trials prior to the study trials.

**Figure 1:**
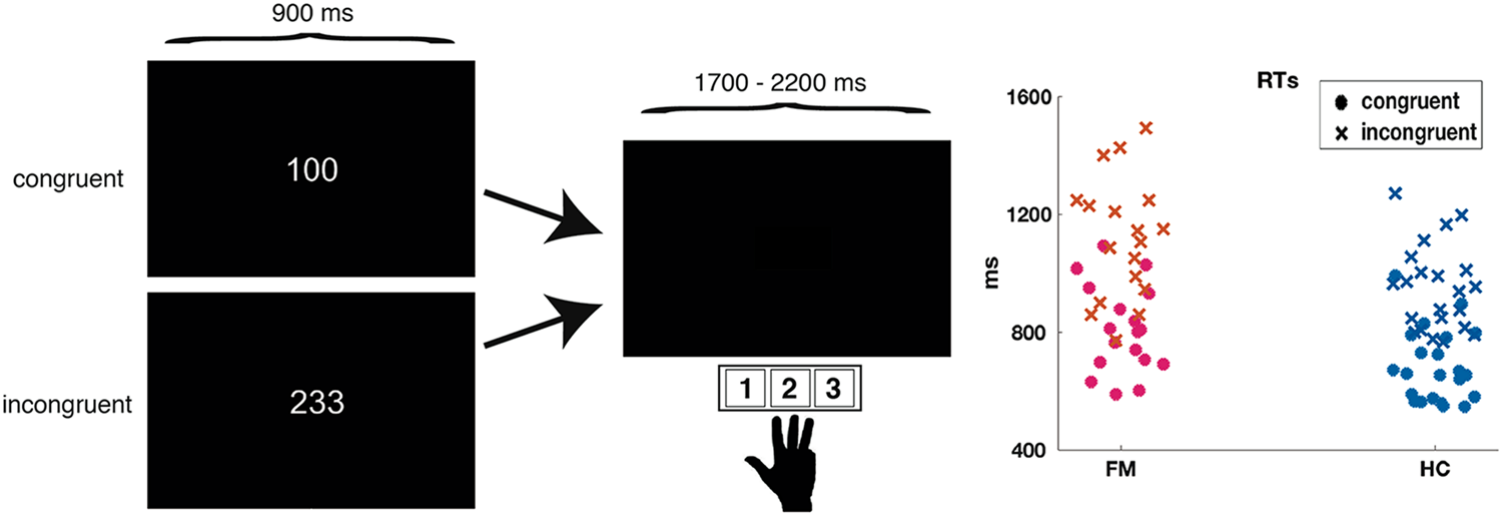
Task design and scatter plot of mean reaction times for each subject in each condition.

The MSIT consisted of 400 randomly presented trials (200 for each congruent and 200 incongruent trials) divided into 10 blocks of 40 trials each. The congruent trials included 3 different types of stimuli (‘100’, ‘020’ and ‘003’), with the different number always located in the congruent stimulus-response position. There were 12 possible combinations in the incongruent condition (‘221’, ‘212’, ‘331’, ‘313’, ‘112’, ‘211’, ‘332’, ‘233’, ‘131’, ‘311’, ‘232’ and ‘322’), and the different number was always located in an incongruent position. Each trial involved presentation of the stimuli for 900 ms followed by an interval during which the screen was black. In order to minimize neural responses related to stimulus expectation, the interval during which the screen was black was varied randomly between 1700 and 2200 ms. Responses were recorded from 150 to 2500 ms after stimulus presentation. After each trial block, participants were allowed to rest for as long as they wanted and they had to press a button in the response box to resume the task (See Fig. 1). The task was designed and presented using PsychoPy^24^.

### EEG recording and analysis

The EEGs were recorded via 62 scalp active electrodes (10–20 international system) inserted in an electrode cap (ActiChamp) and referenced to the tip of the nose. Recordings were made with a BrainVision amplifier (Brain Products inc.). Four additional surface electrodes were placed 1 cm above and below of the right eye and in the lateral canthus of both eyes to monitor vertical and horizontal ocular movements. The FPz electrode was used as ground. Impedances were maintained below 10 kΩ. The EEG signal was sampled at 500 Hz and filtered with a 0.1–100 Hz online band-pass filter and a 50 Hz notch filter.

The EEG data were processed using EEGlab 13.3 toolbox^25^. Data from poorly recording channels were replaced by spherical-spline interpolation. EEG segments containing large ocular or other artefacts, identified by visual inspection, were rejected. Data were filtered with 0.1 Hz high-pass and 80 Hz low-pass FIR filters, and epochs between 800 ms pre-stimulus and 1800 ms post-stimulus were extracted. After removing linear trends of epochs, an extended version of the Independent Component Analysis (ICAs) algorithm was applied, and the components related to ocular or muscular artefacts were manually removed. In order to prevent bias, the experimenter who carried out the manual pre-processing did not know which group each participant belonged to.

The Power Spectral Density (PSD) function of the EEG was calculated for each channel separately using the “spectopo.m” function included in EEGlab. PSD was computed for the period between 200 and 1200 ms after stimulus presentation and for all epochs by using the Welch’s average modified periodogram. One second windows of the EEG with 50% overlap and multiplied by a Hamming window were used. Each window was transformed from the temporal domain to the frequency domain using the Fast Fourier Transform. Then the mean PSD of all scalp electrodes was computed, and a linear regression was fitted to the data in a log-log space (i.e. both power and frequency in a logarithmic scale). The regression was computed for power values between 3 and 30 Hz; higher frequencies were not included because they can be affected by noisy muscular activity. The values of the slope obtained for each participant and condition (i.e. congruent vs. incongruent trials) were used in the statistical comparisons.

A spherical Laplacian spline surface filter was then applied to the EEG data. This filter removes spatially broad features of the data, while leaving local features intact. It is useful for minimizing false connectivity patterns that are due to volume conduction and has been used and validated for this purpose in previous research^26–29^. The surface Laplacian filter was applied using the Matlab code provided by Kayser and Tenke^30^ (with splines of *m* = 4; smoothing constant = 10^−5^).

Time-frequency decomposition was performed by computing the inverse Fast Fourier Transform of the multiplication of the power spectrum of the EEG data by the power spectrum of different complex Morlet wavelets. Wavelets were created in 40 logarithmically frequency spaced steps (from 3 to 80 Hz), with 3 cycles at the lowest frequency up to 8 at the highest frequency, also in logarithmically increasing steps. Event-related spectral perturbation was normalized by transforming the power change of each time-frequency pixel to dB, relative to the mean power in the baseline interval (−400 to −100 ms) for each frequency. The time-frequency power values at FC1, FCz and FC2 electrodes were averaged for midfrontal locations and those at PO3, POz and PO4 were averaged for posterior locations, thus creating 2 virtual electrodes (midfrontal and posterior). The mean theta power (3–6 Hz) change at midfrontal locations and the mean alpha power (9–12 Hz) at posterior locations was computed and used to select time-frequency windows for statistical comparison. Taking into account the long-lasting power modulations in the incongruent condition, we selected 2 different time windows (300–600 ms and 600–1100 ms) for the statistical comparisons. The electrodes were selected on the basis of previous findings relating theta to midfrontal scalp locations and alpha to posterior areas; the power modulation was also clearest at these electrodes. The time and frequency intervals in which the power modulations were most notable were selected after averaging the spectrograms for all participants. This procedure allows selection of time-frequency windows independently of any between-group differences.

Noise can complicate communication of large scale networks, and we therefore analysed the phase connectivity between frontal and posterior areas. We chose these locations because the MSIT is known to activate fronto-parietal cognitive-attentional networks^20^ and also because of previous reports of large scale connectivity between fronto-posterior areas^28^. We thus computed the Phase-Locking Value (PLV) to analyse phase-based connectivity between these scalp areas^31^. This method measures the across-trial consistency of phase differences between two signals. In the present study we averaged the EEGs at FC1, FCz and FC2 electrodes for midfrontal locations and those at PO3, POz and PO4 for posterior locations; use of the average values enabled us to increase the signal/noise ratio. Phase Locking Values between midfrontal and posterior locations were computed for the same time-points and frequencies as used for time-frequency analyses. The values were then baseline corrected by subtracting the mean PLV of each frequency in the period from −400 to −100 ms before stimulus presentation. At this stage, we used the same procedure as before to select time-frequency windows for power. We averaged the connectivity values of both groups and conditions and then selected the time-frequency window with the highest connectivity values. Statistical analyses were applied to the mean PLV values of these windows for each subject and condition.

Groups were compared in relation to socio-demographic values and the VAS and FSQ data by t-tests for independent samples (Table 1). Repeated measures ANOVAs with condition as the within-subject factor and group as the between-subject factor were used to analyse behavioural (reaction times [RTs], standard deviation of RTs and number of correct responses) and electrophysiological data (PSD slope, PLV and alpha and theta power). When appropriate, Greenhouse-Geisser corrections were applied to control for violation of sphericity.

As the difference between the incongruent RT and the congruent RT can be used to determine whether between-group differences are due to difficulties in cognitive control or to alterations in processing speed^32^, we also calculated this index and performed an independent samples t-test to determine any between groups difference.

## Results

### Demographics and characteristics of participants

The groups did not differ in age or years of education (Table 1). For all the remaining variables patients were significantly more affected than controls. Note that patients showed significantly more subjective complaints of cognitive dysfunction (see MFE test, and VAS).

### Behavioural data

Considering RTs, the ANOVA showed significant effects for group (F_(1,38)_ = 9.6; p = 0.004) and condition (F_(1,38)_ = 515.6; p < 0.001), but not for the group*condition interaction (F_(1,38)_ = 2.87; p < 0.09). The FM patients displayed slower RTs than controls (FM: 963 ± 236 ms; HC: 814 ± 148 ms), and RTs for the incongruent condition were slower than for the congruent condition (1022 ± 192 ms vs. 739 ms ± 236). We did not find any statistical significant difference between the FM and HC group in the RT difference (RT incongruent − RT congruent) (t_(23.3)_ = 1.6; p = 0.13; FM: 307 ± 105 ms; HC: 264 ± 50 ms).

One predicted consequence of heightened neural noise is higher standard deviations of reaction times^33^. Although mean standard deviations of RTs were higher for FM in both congruent (FM: 209 ± 80 ms; HC: 164 ± 81 ms) and incongruent conditions (FM: 268 ± 103 ms; HC: 216 ms ± 80 ms), between-group differences were not significant (F_(1,38)_ = 3.25; p = 0.08).

Regarding accuracy, we found a smaller proportion of correct responses in incongruent (0.90 ± 0.12) than in congruent trials (0.98 ± 0.13; F_(1,38)_ = 26; p < 0.001); the group effect was not significant (F_(1,38)_ = 1.9; p < 0.17).

### Power Spectral Density (PSD) slope

**Figure 2:**
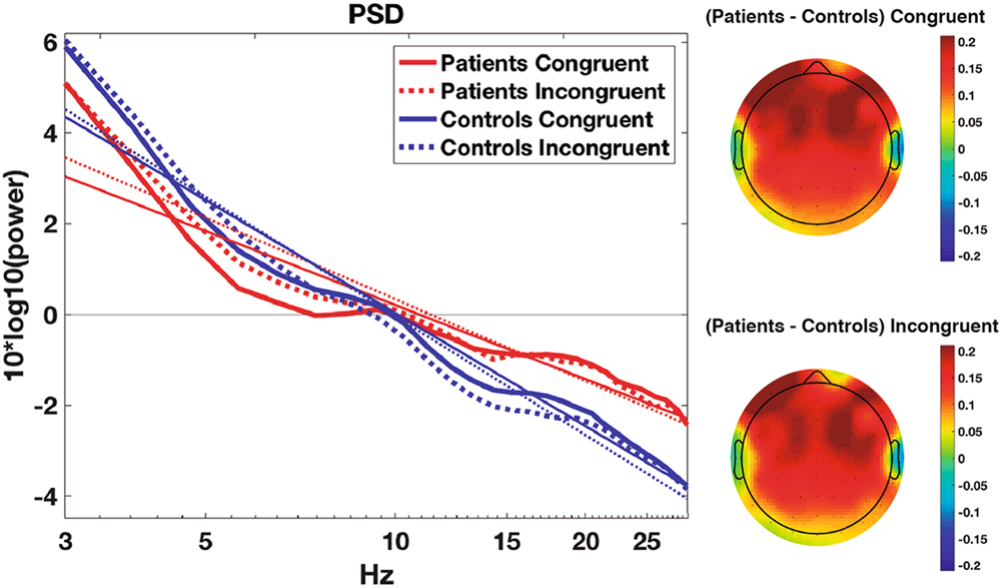
Mean Power Spectral Density (PSD) at all electrodes for each group and condition. Thin lines represent the least squares regression. Topographies show the difference in slope between groups for each condition. As may be seen, the difference between groups in slopes is more evident at frontal locations.

The ANOVA revealed an effect of group (F_(1,38)_ = 7.88; p = 0.008), with a flatter slope for FM patients (−2.78 ± 1.6) than for HC (−4.15 ± 1.5). An effect of condition was also observed (F_(1,38)_ = 16.38; p < 0.001), with steeper slopes for incongruent (−3.6 ± 1.6) than for congruent trials (−3.3 ± 1.5). See Fig. 2.

To ensure that PSD slope was not affected by differences in the contact of the sensors to the scalp, we compared the electrode impedances between groups. The t-test for independent samples showed that there were no differences between groups in the global mean impedance (t_(38)_ = −0.16; p = 0.9; FM: 4.08 ± 1.3 Kohm; GC: 4.13 ± 1.4 Kohm). In addition, there were no between-group differences in the reference impedance values (t_(38)_ = −0.68; p = 0.5; FM: 2.2 ± 2 Kohm; HC: 2.7 ± 2.8 Kohm) or ground electrodes (t_(38)_ = 0.2; p = 0.8; FM: 5.1 ± 1.4 Kohm; HC: 5.0 ± 2 Kohm). These results rule out the possibility that the between-group differences in the PSD slope may be attributed to different quality of the EEG recording.

### Time frequency power modulations

Theta power (3–6 Hz) increased over midfrontal areas after stimulus presentation. In the first time interval studied (300–600 ms), the power increase was greater in HC than in FM patients (F_(1,38)_ = 12.74; p = 0.001; FM: 1.25 ± 0.8 dB; HC: 2.1 ± 0.9 dB). In the later time interval (600–1100 ms), the theta increase was also higher for HC (F_(1,38)_ = 6.5; p = 0.015; FM: 1.63 ± 1 dB; HC: 2.2 ± 0.7 dB). In addition, for these late latencies theta power was higher in incongruent than congruent trials (F_(1,38)_ = 56.9; p < 0.001).

**Figure 3:**
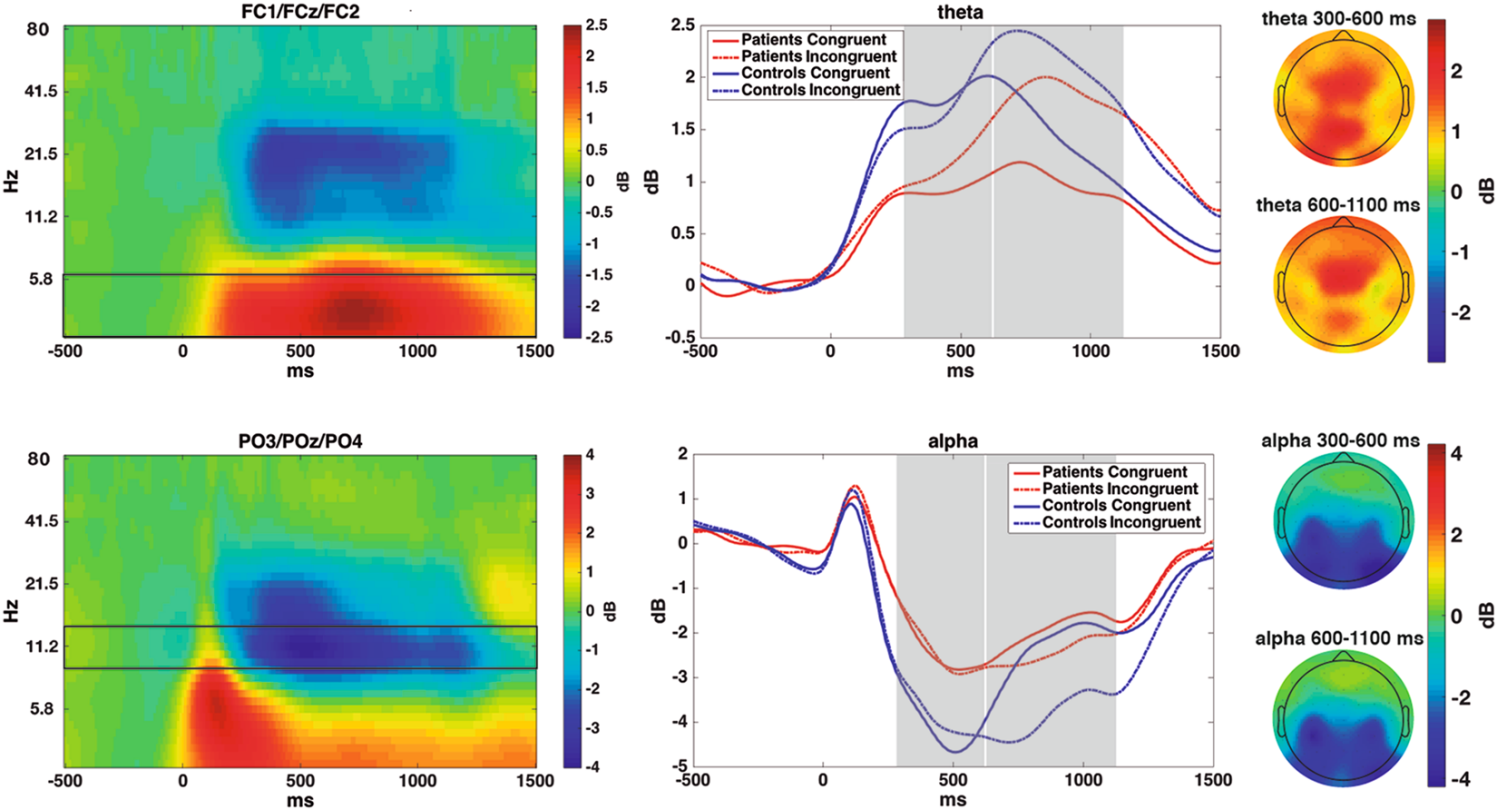
*Left:* Time-frequency power modulations over midfrontal and posterior locations, averaged between groups and conditions. Boxes show the frequency ranges depicted in the left column. *Middle:* Time course of theta and alpha power modulations for each condition and group; Grey boxes show the time windows used for statistical comparisons. *Right:* Topographies -averaged between conditions and groups- for each frequency and time window.

The decrease in posterior alpha (9–12 Hz) in the first time interval measured (300–600 ms) was greater in HC than in FM patients (F_(1,38)_ = 4.5; p = 0.04; FM: −2.5 ± 2.2 dB; HC: −4.2 ± 2.6 dB). For later latencies (600–1100 ms), significant main effects of condition (F_(1,38)_ = 22.2; p < 0.001) and group*condition interaction (F_(1,38)_ = 5.6; p = 0.02) were observed. Post-hoc comparisons revealed differences in alpha power between conditions for the control participants (p < 0.001), but not the FM patients (p = 0.12). See Fig. 3.

### Distant-range functional connectivity

**Figure 4:**
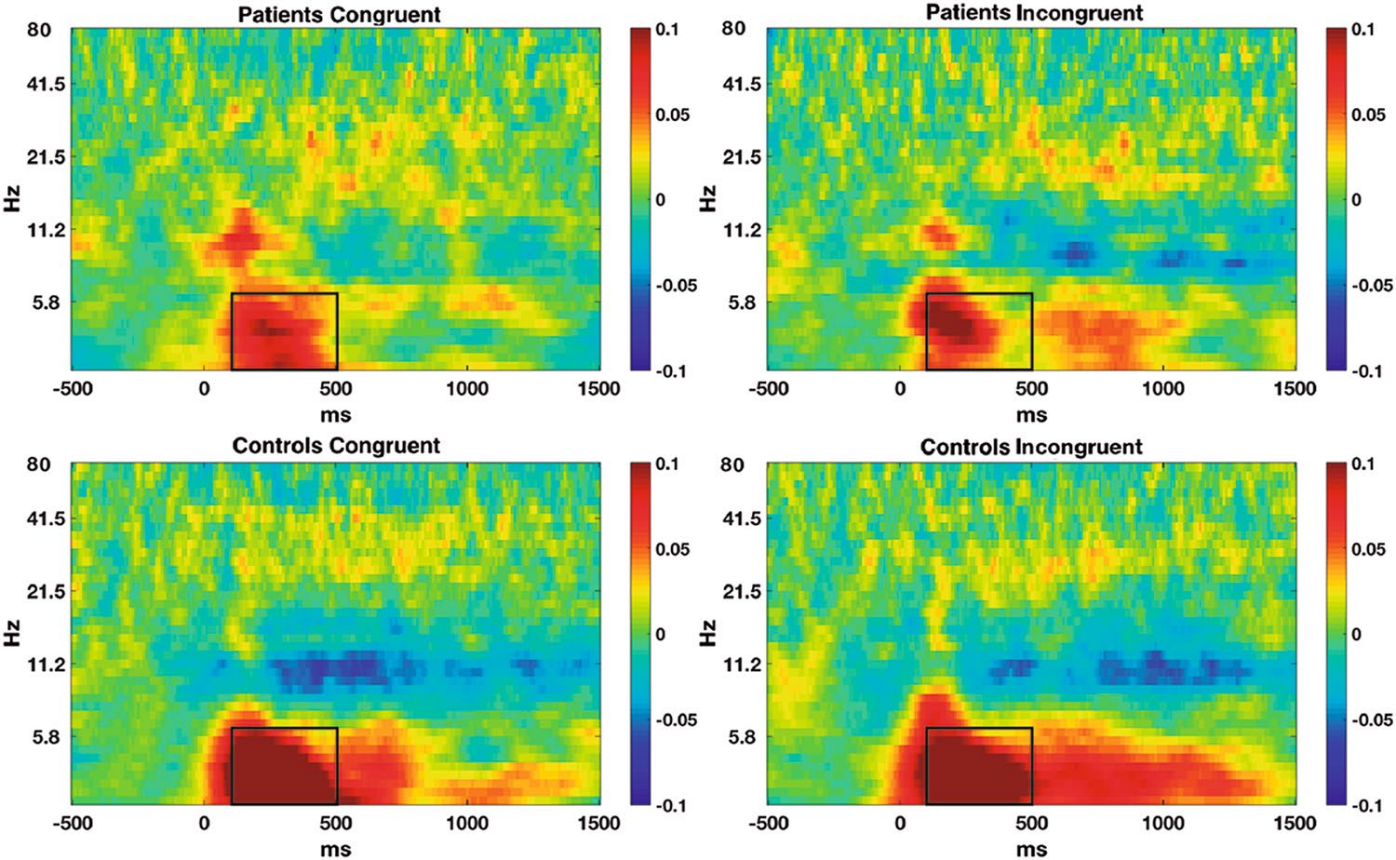
Phase-locking values between midfrontal and posterior locations for each group and condition. Boxes show the windows used for statistical comparisons.

We observed a general increase in phase synchronization from 100 to 500 ms and from 3 to 6 Hz. ANOVA of PLV values in this window revealed a significant group effect, with lower phase synchronization in FM patients than in HC (F_(1,38)_ = 7.59; p = 0.009; FM: 0.06 ± 0.06; HC: 0.11 ± 0.08). No significant effects for condition or group*condition interaction were observed. See Fig. 4.

## Discussion

Fibromyalgia and other chronic pain patients frequently report cognitive and attentional impairments. Although the neural noise hypothesis may provide an explanation for such alterations, this phenomenon has not previously been investigated in chronic pain syndromes. In the present study we observed flatter PSD slopes for FM patients than for healthy controls. In addition, FM was related to reduced posterior alpha and midfrontal theta power modulation, as well as to reduced fronto-posterior theta phase synchronization during performance of a task that activates cognitive/attentional networks. The data corresponding the three EEG indices considered are consistent and suggest a link between FM and heightened neural noise.

The human brain is a noisy environment in which random intrinsic electrical activity occurs. As cognition depends on effective neural communication, excessive levels of noise may complicate information processing^34^. A way of determining the level of noise in the brain is to measure the slope of Power Spectral Density (PSD) plots^10^. EEGs with highly uncorrelated and random activity yield flatter PSD slopes, while more organized and predictable systems yield steeper slopes^10, 11, 13^. In the present study we observed flatter slopes for chronic pain patients than for healthy controls. Topographical representations show that these differences are greater at frontal locations (see Fig. 2). Between-groups differences in PSD slopes were observed in both congruent and incongruent task conditions, suggesting that the higher levels of noise in chronic pain patients are present during both lower cognitive load and increased cognitive demand periods. As mentioned in the results section, between-group differences in PSD slope are unlikely to be due to different quality in the recording of the EEG. The differences are also probably not due to muscular artefacts, as characteristic muscular activity appears at temporal locations, while the observed slope differences are maximal at frontal scalp locations.

Additionally, we found that incongruent trials had steeper slopes than congruent ones. In this respect, situations that require higher cognitive control are related to higher theta power, a brain rhythm that coordinates the spiking activity of single neurons^35^, and consequently, if neural populations are activated in a more synchronized way -with less aberrant neural activity- the PSD slope is expected to be steeper^15^. Therefore, finding a more pronounced slope for incongruent trials is consistent with the coordinating role of theta oscillations.

Alterations in PSD slope may be caused by changes in the neural excitatory/inhibitory ratio^36^, which are mainly created by respectively glutamatergic and GABAergic synaptic inputs, and are crucial for generating oscillatory activity^37^. Gao *et al*.^14^ suggested that flatter PSD slopes are associated with greater excitation and/or reduced inhibitory activity. Thus, our results are consistent with reports of downregulation in GABA and upregulation in glutamate routes for FM and other chronic pain pathologies^38–40^. A reduction in the PSD slope has also been observed in aging and its related cognitive decline^11^. In connection with this, it was suggested that fibromyalgia patients may show a premature brain aging, with increased grey matter loss for their age^41, 42^.

Although noise usually degrades the transmission of information, it can have the opposite effect under certain conditions -such as stochastic resonance processes-^43^. Stochastic resonance enables enhancement of the detection and transmission of weak signals by introducing certain levels of noise in threshold-like systems^34^. The addition of noise increases the probability that a weak signal, which would not cross the threshold in a noise free environment, will reach the detection threshold. In this respect, the enhanced detection of pain (hyperalgesia) observed in fibromyalgia and other pain patients^44, 45^ may be explained by stochastic-resonance-type effects. Although this appears to be supported by the PSD slope data, further research is needed to confirm these hypothesis.

We also expected that noise should be related to impaired firing correlation in local networks, and therefore analysed midfrontal theta and posterior alpha waves, two prominent rhythms that coordinate local and large scale networks and interact between them^28^. In congruence with previous reports^46^, we found that FM patients showed a lower increase in theta power after stimulus presentation. In addition, only healthy controls maintained the enhanced theta power of the incongruent condition at later latencies. Midfrontal theta oscillatory activity is functionally related to cognitive control and top-down attentional modulation^17, 47^. In this sense, the observed group differences in theta power may be explained by higher difficulties of FM patients in mobilizing and retaining attentional resources. We also observed a smaller decrease in alpha power in the FM patients than in healthy controls. Posterior alpha oscillations are related to rhythmic fluctuations of cortical inhibition of task-irrelevant brain areas^16^. Therefore the less sustained reduction of alpha power in pain patients may be explained by impaired cortical implication of posterior brain locations during performance of the MSIT.

Theta oscillations are assumed to coordinate large scale networks by modifying the firing probability of distant neurons and by recruiting additional brain areas under cognitive-demanding conditions^17^. We observed reduced theta phase synchronization between frontal and posterior locations in FM patients. As cognition is dependent on dynamic interactions between distant brain areas, and given that the MSIT activates the fronto-parietal network, this finding also provides neurophysiological evidence for cognitive-attentional dysfunction in FM patients, suggesting some type of functional disconnection between attentional control-related frontal networks and perception-related posterior networks^48^. Considered together, the results indicate higher levels of neural noise in chronic pain patients than in the controls.

In other study involving performance of the MSIT by FM patients, Veldhuijzen *et al*.^32^ found that the RT difference (incongruent - congruent) was similar in patients and controls, concluding that there were no alterations in cognitive control or inhibitory processes in the patients. The results obtained here for behavioural performance led to the same conclusion. Nevertheless, the group differences in midfrontal theta power for the incongruent condition (especially at late latencies) suggest that cognitive control may also be altered in FM.

### Limitations

Participants were asked not to consume more drugs than necessary, but prescribed medication (mainly antidepressants and anxiolytic drugs) was not withdrawn. Therefore the obtained results may be affected by the medication taken by patients. In any case, the patients are thus representative of the general chronic pain population (usually under various pharmacological treatments). Additionally, the various medications may have positive or negative effects on cognitive functioning^49^. Finally, temporary drug withdrawal could induce negative effects on cognitive function or alterations in brain activity that would also complicate interpretation of the data.

Although the use of the Laplacian spatial filter is useful to eliminate spurious functional connectivity, it can introduce noisy activity in the EEG^50^. To check the reliability of the data, we compared our time-frequency results with the obtained after using other re-referencing technique (i.e. REST^51^). We observed the same significant differences in time-frequency data either using the spherical Laplacian or the REST technique, suggesting that the reported results are robust.

## Conclusion

In conclusion, we provide convergent results that suggest higher levels of neural noise in FM patients and that such alterations may explain the dyscognition reported by these patients. This is the first attempt to relate chronic pain to neural noise, and further research should clarify how noise-related factors (such as stochastic resonance) affects hypersensitivity in chronic pain. This new explanation has clinical implications as it suggests that treatments focused on reducing noise and increasing neural synchronization may benefit chronic pain patients.
